# WATCH AFib: smartwatches for detection of atrial fibrillation in secondary prevention of cryptogenic stroke—protocol for a prospective, intraindividual-controlled, multicentre clinical study

**DOI:** 10.3389/fneur.2025.1661087

**Published:** 2025-11-06

**Authors:** Horst Penkert, Johanna Härtl, Alexander Hapfelmeier, Silvia Egert-Schwender, Edith Heimsch, Sabine Friedenberg, Alexander Müller, Franziska Hahn, Eimo Martens, Silke Wunderlich

**Affiliations:** 1 Department of Neurology, TUM University Hospital, School of Medicine and Health, Technical University of Munich, Munich, Germany; 2 Institute of AI and Informatics in Medicine, TUM University Hospital, School of Medicine and Health, Technical University of Munich, Munich, Germany; 3 Muenchner Studienzentrum (MSZ), School of Medicine and Health, Technical University of Munich, Munich, Germany; 4 Department of Internal Medicine I, Cardiology, TUM University Hospital, School of Medicine and Health, Technical University of Munich, Munich, Germany

**Keywords:** ischemic stroke, transient ischemic attack, atrial fibrillation, smartwatch, rhythm monitoring, telemedicine

## Abstract

**Rationale:**

Detection of atrial fibrillation (AFib) and subsequent anticoagulation therapy reduce the risk of recurrent stroke, while prolonged rhythm monitoring significantly increases AFib detection. Thus, prolonged smartwatch-based ECG monitoring after cryptogenic ischemic stroke or transient ischemic attack (TIA) could lead to a reduction of recurrent stroke by prompting adequate anticoagulation therapy.

**Aim:**

WATCH AFib investigates the accuracy of smartwatches for AFib detection in patients with cryptogenic TIA or ischemic stroke compared to an implantable event recorder.

**Sample size:**

40 cases of AFib are required to estimate the sensitivity for AFib detection per patient with a precision of about 10%. As AFib is observed in 9%−16% of cryptogenic strokes, we intend to enroll 400 patients.

**Methods:**

WATCH AFib is a prospective, intraindividual-controlled, multicentre clinical study in patients with cryptogenic ischemic stroke or TIA. ECG-data from smartwatches and event recorders is continuously monitored by two independent cardiologists for a follow-up period of 6 months. If AFib is detected, therapeutic options are discussed at the including center.

**Primary outcome:**

To compare smartwatch- and event recorder- based sensitivity and specificity of AFib detection per patient after 6 months.

**Discussion:**

Prolonged AFib screening after stroke is currently suboptimal. Smartwatches might be a non-invasive, cost-effective, widely available alternative for prolonged rhythm monitoring. Usability in severely affected patients and patients with persisting neurological deficits might be limited.

**Trial registration:**

The study is registered on clinicaltrials.gov. Registration number: 20230726.

## 1 Introduction

In the secondary prevention of ischemic stroke, detection of atrial fibrillation (AFib) and subsequent anticoagulation therapy reduce the risk of recurrent stroke by approximately 60% (1, 2). Cryptogenic stroke is defined as ischemic stroke for which no probable cause is found despite a full standard evaluation and comprises 25% of all ischemic strokes (3). Prolonged electrocardiogram (ECG) monitoring for 30 days to 6 months significantly increases detection of occult paroxysmal AFib, which is present in 9%−16% of cryptogenic strokes (4–7). Careful patient preselection can increase detection rates up to 28% in 6 months (8). A meta-analysis of 1,102 patients revealed that prolonged ECG monitoring after ischemic stroke correlates with higher detection rates of paroxysmal AFib, initiation of anticoagulant therapy, and decrease of stroke recurrence (9). Thus, prolonged ECG monitoring is likely to lead to a reduction of recurrent stroke by prompting adequate anticoagulation therapy.

Still, the most efficient and cost-effective way of rhythm monitoring after a cryptogenic stroke is unclear (10–12). In addition, prolonged AFib screening using implantable cardiac monitors (ICM) is currently suboptimal due to a limitation of resources, loss to follow-up, invasiveness of procedures, and costs (13). A fast growing body of evidence in the field of cardiology states that smartwatches detect AFib with similar sensitivity and specificity to other wearable devices (i.e., Holter- ECGs and others) and ICMs. Continuous derivation of photoplethysmography (PPG)- signals by a smartwatch has been shown to sufficiently detect AFib in the general population with a positive predictive value of 0.84–0.98 (14–16). Similarly, comparing PPG-based to in hospital ECG-based diagnosis, sensitivity and specificity of AFib detection is high (93%−98% and 90%−98%, respectively) (17–19). Compared to implantable cardiac monitors, PPG- based AFib diagnosis exhibits a sensitivity of 97.5% for AFib episodes >1 h and a sensitivity of 100% for AFib detection per patient (18). Current generations of smartwatches are further able to perform a patient activated one-lead ECG, which shows good sensitivity (93.5, 94.4%) and specificity (100, 81.9%) compared to 12-lead ECG monitoring (20, 21) and may enhance diagnostic accuracy by combination of both methods. Nevertheless, a high number of inconclusive recordings after automated analysis (20%−30%) constitutes a limitation, and can be overcome by cardiologist review (20–22).

Recently, it could be shown that patient acceptance of smartwatches (Pulsewatch) for AFib detection in stroke patients >50 years of age is high (23, 24). Up to now, only one study compares smartwatches to ECG patches for 14 + 30 days in stroke survivors and concludes that smartwatches are feasible for long-term arrhythmia monitoring. Nevertheless, as AFib was detected in only five patients, and the follow-up period was relatively short, the results of the study are preliminary (24). Further studies are urgently needed to address the existing lack of evidence.

As stroke patients often suffer from relevant disabilities, a transfer from the hitherto existing data about AFib and smartwatches from otherwise healthy individuals cannot be assumed. To our knowledge, there is only one other ongoing study assessing the accuracy of smartwatch-derived PPG signal for AFib detection in a stroke population under real-life circumstances (NCT05006105). So far, the protocol or final results have not been published.

We hypothesize that AFib detection via smartwatch in patients suffering from cryptogenic transient ischemic attack (TIA) or ischemic stroke is accurate for AFib detection compared to an implantable Event Recorder and hence introduce the following prospective multi-center clinical trial (WATCH AFib). If our hypothesis proves right, smartwatches might be a non-invasive, cost-effective, widely available alternative for rhythm monitoring, which could potentially change the current standard of post-stroke care.

## 2 Methods and analysis

### 2.1 Study design

The clinical study is carried out in accordance with the study protocol and the principles of the Declaration of Helsinki by the World Medical Association and specific applicable national ethical and regulatory requirements. The study was approved by the local ethics committee.

**Figure 1:**
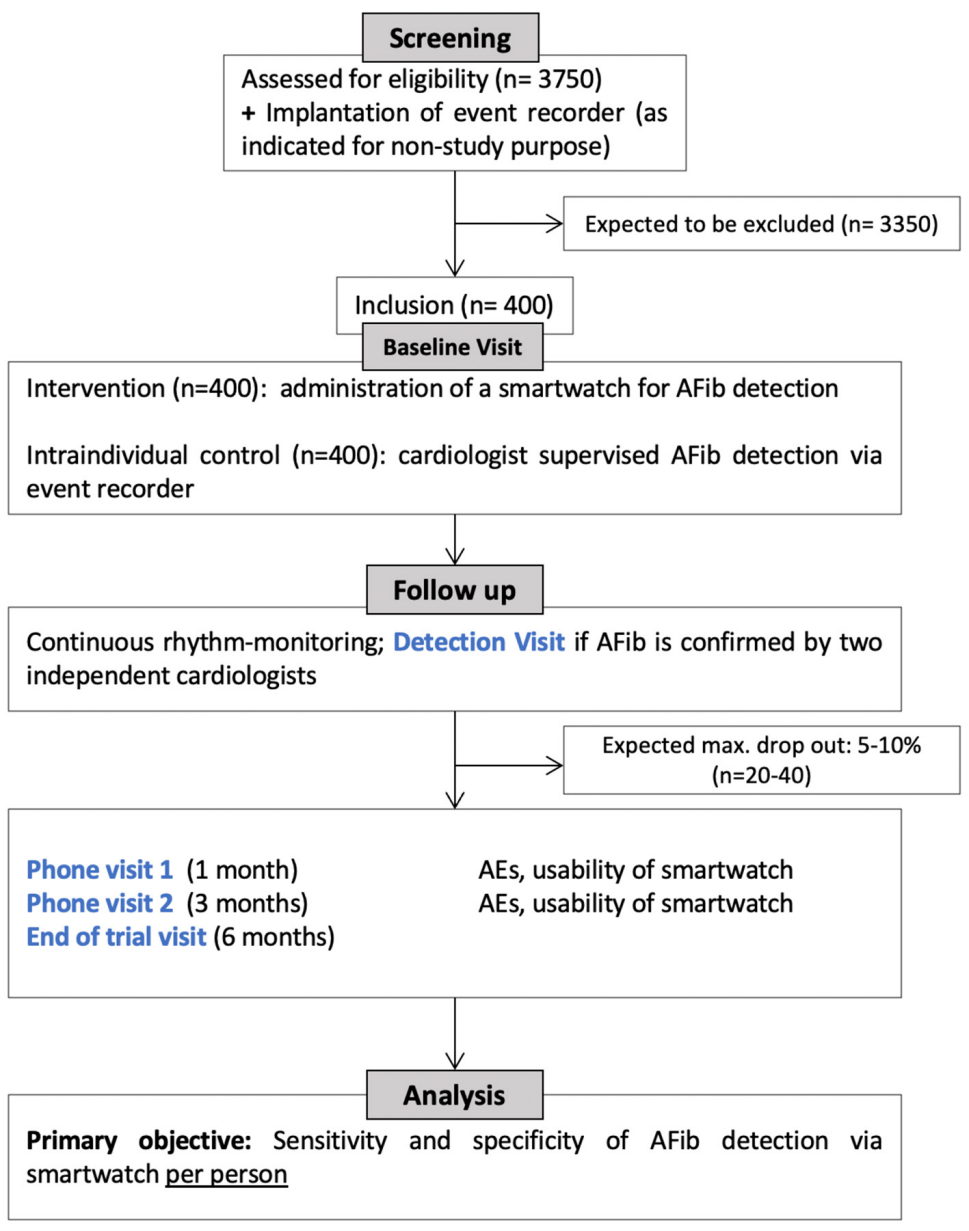
Study flowchart.

We conduct a prospective, intraindividual-controlled, multicenter clinical study, as depicted in Figure 1. The study population includes patients with cryptogenic TIA or ischemic stroke and known risk factors for the presence of paroxysmal AFib (see inclusion criteria). As participants are recruited multi-centric and nationwide, it is expected that results are representative for the German/European ischemic stroke population and are transferable to the general population with TIA or ischemic stroke. As no strong recommendation on the selection of stroke patients for event recorder implantation exists, and as the event recorder is not part of the study intervention, we do not specify selection criteria for implantation.

Patient screening and inclusion are performed at selected study centers according to the defined inclusion and exclusion criteria (see below). Standardized onboarding training, printed guides, and caregiver assistance documentation are provided for all study sites. The study centers hold cross-regional stroke units treating >1,000 patients with ischemic stroke or TIA per year and collaborate with cardiologists experienced in implantation of event-recorders. All patients included in the clinical study will receive standard of care for cryptogenic TIA/ischemic stroke. The study intervention consists of wearing a smartwatch (i.e., Scanwatch, Withings), which is CE-certified as medical device for AFib detection. Smartwatch- and Event Recorder-derived heart rhythm are daily uploaded and analyzed by cardiologists at the Cardiology Core Lab at the Department of Internal Medicine I, TUM Klinikum Rechts der Isar, Munich. Obtained data on cardiac arrhythmia will be accessible for each study site in consultation with the Cardiology Core Lab. In case of AFib detection (defined as arrhythmia lasting longer than 30 s), the including study site will be informed to set an *ad-hoc* detection visit and determine further therapeutic options. The study also includes a total of four pre-scheduled visits, to confirm eligibility, to record stroke and patient characteristics, smartwatch application and usability, stroke recurrence and adverse events (see Table 1). The baseline visit (Visit 0) may take place within the clinical setting of the acute stroke work up. The second and third visit (Visit 1, Visit 2) constitute phone-visits at 1 and 3 months. The last visit at the end of the study (Visit 3) will be performed at 6 months. In-patient visits will take place at each patient's study center.

**Table 1:** Data to be collected.

**Data to be collected**	**Visit 0**	**Visit 1^¥^**	**Visit 2^¥^**	**Visit 3**	**Detection visit**
	**Max. 6 months after stroke/TIA**	**1 month** ±**1 week**	**3 months** ±**1 week**	**6 months** ±**3 weeks**^#^	**If AFib is confirmed**
In-/exclusion criteria^a^	X				
Patient characteristics^b^	X			X	
Stroke characteristics^c^	X				
Physical examination^d^	X			X	
NIHSS	X			X	
mRS	X	X	X	X	
Application of smartwatch^e^		X	X	X	
Stroke/TIA recurrency		X	X	X	
AESI/device incidents		X	X	X	
Detection of AFib^f^					X
Study termination				X	

^a^See methods.

^b^Includes age, sex, medical history, and cardiac risk factors on echocardiography (TTE/TEE).

^c^Includes date of index event, vascular occlusion and site of occlusion, application of intravenous thrombolysis or mechanical thrombectomy, NIHSS at initial presentation, premorbid mRS, mRS at initial presentation, discharge.

^d^Includes date of examination, impaired hand-/arm function on the dominant limb, neglect, hemianopsia, apraxia, aphasia.

^e^Includes hours of daily application, nightly application, comfort, user friendliness.

^f^Date of visit, visit location, evaluation of oral anticoagulation and possible further medical/interventional treatment.

^¥^Phone visit.

^#^Or in case of premature termination of the study.

#### 2.1.1 Inclusion criteria

- Event recorder with telemedicinal function, implanted at the discretion of the attending physician.
- Cryptogenic stroke, or TIA with definite cortical syndrome (aphasia, neglect or homonymous hemianopia) within the last 6 months after full standard evaluation:
  - Stroke detected by computed tomography (CT) or magnetic resonance imaging (MRI) that is not lacunar (lacunar is defined as a subcortical infarct in the distribution of the small, penetrating cerebral arteries whose largest dimension is ≤ 1.5 cm on CT or ≤ 2.0 cm on MRI diffusion images),
  - Absence of extracranial or intracranial atherosclerosis causing ≥50 percent luminal stenosis of the artery supplying the area of ischemia,
  - No major-risk cardioembolic source of embolism (i.e., no permanent or paroxysmal atrial fibrillation, sustained atrial flutter, intracardiac thrombus, prosthetic cardiac valve, atrial myxoma or other cardiac tumors, high-grade mitral valve stenosis, recent (within 4 weeks) myocardial infarction, left ventricular ejection fraction < 30 percent, valvular vegetations, or infective endocarditis),
  - No other specific cause of stroke identified (e.g., arteritis, dissection, migraine, vasospasm, drug abuse),
  - No paroxysmal atrial fibrillation in 72 h of in-hospital ECG-monitoring, including at least one Holter- ECG for 24 h.
- Age: ≥40 years.
- At least one of the following risk factors:
  - CHA2DS2VASc score ≥4,
  - Atrial runs ≥20 consecutive supraventricular premature beats,
  - Left atrial size > 45 mm,
  - Left atrial appendage flow ≤ 0.2 m/s.
- No contraindication for anticoagulant therapy after acute phase of stroke.
- Written informed consent by patient or authorized caregiver.

#### 2.1.2 Exclusion criteria

- Patient is not able to perform one-lead ECG recording with smartwatch,
- Patient possesses no smartphone (iOS version 15.0 or later; Android 9 or later),
- Implanted pacemaker or cardioverter defibrillator (ICD),
- Pregnancy or breastfeeding period.

### 2.2 Objectives

#### 2.2.1 Primary objectives

The primary objective is to assess whether AFib detection via smartwatch in patients suffering from cryptogenic TIA/ischemic stroke is accurate in comparison to implantable event recorders. This will be assessed by sensitivity and specificity of AFib detection per patient after 6 months. We aim on a comparison of smartwatch based, continuous, automated, cardiologist supervised rhythm analysis of photoplethysmography (PPG)- signal and patient activated one-lead ECG with event recorder based, continuous, automated ECG rhythm analysis.

#### 2.2.2 Subgroup analyses

As smartwatch usability might be impaired for patients with residual neurological deficits or severely affected patients, we predefine the following analyses for this clinically relevant subgroups:

- Sensitivity and specificity for AFib detection in patients with residual neurological deficit (aphasia, apraxia, hemianopsia, neglect, or a hemiparesis on the dominant extremity)
- Sensitivity and specificity for AFib detection in severely affected patients (i.e., NIHSS ≥8).

#### 2.2.3 Secondary objectives

- Positive and negative predictive values for AFib detection per patient within 6 months
- Sensitivity and specificity for AFib detection per patient based on automated PPG-signal rhythm analysis
- Sensitivity and specificity for the detection of any AFib episode
- Sensitivity for the detection of AFib episodes >1 h
- Specificity for episodes of sinus rhythm >1 h
- Sensitivity and specificity of AFib detection per recorded/per analyzable time (i.e., intervals in which the watch is actually worn/records an analyzable signal)
- Ischemic stroke and TIA recurrence within 6 months.

#### 2.2.4 Exploratory objectives

Exploratory objectives include the acceptance and practicability of smartwatches for AFib detection (assessment of patient responses via self-designed questionnaire with ordinal items), AFib burden/ patient, AFib risk factors, AFib detection rates after 1, 3, and 6 months, time to confirmed AFib diagnosis, and count of AFib diagnoses.

### 2.3 Data monitoring body

The Muenchner Studienzentrum (MSZ), an independent clinical research institution at the School of Medicine and Health, Technical University Munich, is responsive for quality assurance. Monitoring activities are performed to ensure that the study is conducted in accordance with the protocol.

### 2.4 Sample size estimation

According to a previous study a sensitivity and specificity for AFib detection per patient of >99 and 90%, can be assumed, respectively (18). Thus, 40 cases of AFib are required to estimate the sensitivity with a precision of about 10%, i.e., the difference of the lower bound of a two-sided exact 95% confidence interval and the point estimate of the sensitivity is 10%. As other studies observe paroxysmal AFib in 9%−16% of patients with cryptogenic stroke (4, 5), we intend to enroll 400 patients to obtain the required 40 cases. The specificity can consequently be measured with a precision of 4%. Our inclusion criteria should lead to a preselection of candidates with increased AFib risk factors. Therefore, AFib detection rate might increase up to 28% (max = 112) in our cohort within 6 months (8). The event of a 10% drop-out rate (e.g., due to withdrawal of consent, eligibility violations, no intervention, or non-existent data after inclusion into the study) would result in a minimum of 36 AFib cases and a similar precision of estimation i.e., 12% for the sensitivity and 4% for the specificity.

### 2.5 Statistical methods and analysis populations

Point estimates and two-sided exact 95% confidence intervals will be computed for the sensitivity and specificity referring to the primary objectives, related secondary objectives and subgroup analyses. Cardiologist-supervised event recorder-based continuous ECG rhythm analysis serves as the gold standard. Thus, in the case of discordant results, we consider the analysis of the signal from the event recorder by the cardiologist as the true status. As power calculation is based on participants with detected AFib as effective sample size (n_AFib_), we intend on analyzing our data as soon as one of the following situations occurs: (1) AFib is detected in 40 participants, who have completed follow-up or (2) enrolment and follow up is complete for all 400 participants (n_Enrolment_). In the first situation, the study leadership, the statistician, and the steering committee will decide on continuation of enrolment. Exploratory hypothesis testing of comparisons will be performed by McNemar's Chi-squared Test at two-sided 5% levels of significance. Due to the exploratory character of the analysis, and in accordance with the calculation of 95% confidence intervals, there will be no correction for multiple testing. With reference to the ICH E9 Guideline, the analyses will be performed using a full analysis set (FAS), according to the intention-to-treat principle (ITT), and a per protocol set (PP). The former will include all times with event recorder measurements (until possible but unexpected losses to follow-up) and the latter will include only times with additional smartwatch recordings. Therefore, missing values, possibly due to non-compliance, will be rated as no signal of an AFib by the smartwatch in the FAS. The FAS and PP analysis populations coincide when smartwatch records are available in all patients. Patients with eligibility violations (concerning inclusion or exclusion criteria), no intervention or non-existent data after inclusion into the study will be excluded from analysis. Time to confirmed AFib diagnosis, count of AFib diagnoses, AFib burden, safety endpoints and usability of smartwatches will be reported by descriptive statistics (mean, standard deviation, median, interquartile range, absolute and relative frequency). AFib risk factors will be compared between patient groups with and without AFib using descriptive statistics, hypothesis testing and multiple logistic regression models. Pre-defined subgroup analyses will be performed as described above.

## 3 Discussion

Adequate work-up of cryptogenic stroke and especially AFib screening is highly relevant for secondary stroke prevention and risk reduction (1, 2). Naturally, probability of AFib detection correlates with duration and intensity of rhythm monitoring (4, 5). Prolonged AFib screening after stroke is currently suboptimal due to a limitation of resources, loss to follow-up, invasiveness of procedures, and costs.

Application of smartwatches has been shown to sufficiently detect paroxysmal AFib in the general population (14–16). As stroke patients often suffer from relevant disabilities, a transfer from existing data from otherwise healthy individuals cannot be assumed and prospective clinical research is urgently needed. We hence propose the prospective, multi-center clinical study “WATCH AFib” to assess the accuracy of smartwatches for AFib detection in stroke patients.

We choose an intraindividual control for AFib detection and use cardiologist supervised implanted event recorders as gold standard. This does (i) guarantee an accurate evaluation of the sensitivity and specificity and (ii) ensures that all participants receive appropriate diagnostics and subsequent anticoagulation therapy if indicated.

As the study intervention consists in simply wearing a smartwatch, as phone visits are implemented at 1 and 3 months, and as the time of follow-up (i.e., 6 months) is rather short, we expect a low rate of compliance issues or of loss to follow-up and estimate this to be around 5%−10%.

Inclusion criteria were specified for known AFib risk factors to increase detection rates and thus, study power (8). Therefore, the resulting cohort might be older and more diseased than the general stroke population. If smartwatches prove applicable for AFib detection in our cohort, results should be extendable to the general stroke population. In addition, we assess the accuracy of smartwatches in subgroups of patients with persisting neurological deficits or severely affected patients and include patients with authorized caregivers. For technical reasons, patients with pacemakers/defibrillators, patients without smartphones, and participants who cannot actively perform a one-lead ECG using the smartwatch need to be excluded.

Naturally, our study shows some limitations and might face certain obstacles: First, as mentioned above we cannot include patients without smartphones or patients who are not able to actively perform a one-lead ECG. Second, recruitment might be challenging due to limited event recorder implantation in the general stroke population, due to the invasiveness and costs of the procedure. Nevertheless, validation of a new diagnostic tool should be done in comparison to the most accurate, available diagnostic test.

### 3.1 Summary and conclusion

The current study prospectively validates accuracy of smartwatches for AFib detection in patients with cryptogenic stroke or TIA. Smartwatches might be a non-invasive, cost-effective, widely available alternative for prolonged rhythm monitoring, including implantable event recorders, and could potentially change the standard of post-stroke care.
